# Serum human alpha-lactalbumin as a marker for breast cancer.

**DOI:** 10.1038/bjc.1990.173

**Published:** 1990-05

**Authors:** E. T. Thean, B. H. Toh

**Affiliations:** Department of Pathology and Immunology, Monash Medical School, Prahran, Victoria, Australia.

## Abstract

**Images:**


*Br. J. Cancer* (1990), **61**, 773–775 © Macmillan Press Ltd., 1990

### Serum human α-lactalbumin as a marker for breast cancer

E.T. Thean & B.H. Toh

*Department of Pathology and Immunology, Monash Medical School, Commercial Road, Prahran 3181, Victoria, Australia.*

**Summary** Serum levels of α-lactalbumin were assayed using a monoclonal antibody specific for this breast specific molecule. Elevated levels were found in 87% (48/55) of sera from women in the third trimester of pregnancy (29.1 ± 7.4 ng ml$^{-1}$), from 64% (62/97) of patients with breast cancer (23.4 ± 5.6 ng ml$^{-1}$), and from 70% (56/80) of patients with gynaecological cancers (19.4 ± 6.7 ng ml$^{-1}$). These α-lactalbumin levels were significantly higher ($P<0.001$) than those for men and non-pregnant women (11.0 ± 2.3 ng ml$^{-1}$) and for patients with other, non-gynaecological cancers (13.4 ± 3.6 ng ml$^{-1}$). The α-lactalbumin levels were higher in patients with stage IV breast cancer than those with stage I–III breast cancer. The overall sensitivity and specificity of the radioimmunoassay were 72% and 75% respectively. These findings suggest that the assessment of serum levels of α-lactalbumin may be useful as a marker for monitoring breast cancer.

Human α-lactalbumin, comprising 123 amino acid residues (Findlay & Brew 1972), is a modifier protein of galactosyl transferase, an enzyme involved in lactose production. Since the human α-lactalbumin is breast restricted, it is uniquely placed as a breast marker. Indeed, experiments with rodent mammary tumours have shown that this is the case (Schultz & Ebner, 1977*a*; Zamierowski & Ebner, 1980). However, previous attempts to exploit human α-lactalbumin as a marker for human breast cancer using polyclonal rabbit antisera have been unsuccessful (Kleinberg, 1975; Schultz & Ebner, 1977*b*; Woods & Heath, 1977). This is probably due to the presence of 'natural' antibodies to bovine α-lactalbumin which cross react with human α-lactalbumin (Woods & Heath, 1978). These natural antibodies, which have a higher affinity for bovine α-lactalbumin than for human α-lactalbumin (Zangerle & Franchimont, 1985), may have arisen through dietary exposure to cow's milk.

To overcome these problems, we have produced a murine monoclonal antibody which binds specifically to human α-lactalbumin and which does not cross-react with bovine α-lactalbumin (Thean & Toh, 1989*a*,*b*). The present report describes the application of the monoclonal antibody in a radioimmunoassay carried out in the presence of bovine α-lactalbumin, to release human α-lactalbumin pre-bound to the 'natural' antibodies.

### Materials and methods

### *Human α-lactalbumin*

The human α-lactalbumin (Calbiochem, USA) used for the production and characterization of the monoclonal antibody was also used as a standard for the RIA. The purity of the protein was confirmed by two-dimensional (IEF/PAGE) gel electrophoresis (O'Farrell, 1975).

### *Monoclonal antibodies*

The murine monoclonal antibody ET-1 binds specifically to human α-lactalbumin and does not cross-react with bovine α-lactalbumin or bind to human albumin, casein or other milk proteins as assessed by ELISA, Western blotting and immunoprecipitation. Furthermore, the specificity of the antibody for human α-lactalbumin was confirmed by specific inhibition of binding by human α-lactalbumin but not by bovine α-lactalbumin, human albumin or casein (Thean & Toh, 1989*a*,*b*).

Correspondence: E.T. Thean.
Received 22 September 1989; and in revised form 7 November 1989.

### *Patients*

Sera were collected from patients with benign and malignant neoplasms attending the Alfred Hospital, Melbourne, from healthy blood donors (Red Cross Blood Bank, Melbourne) and from women in the third trimester of pregancy (Royal Women's Hospital, Melbourne). All sera were coded and stored at −20°C. Clinical parameters, such as extent of disease were provided by staff clinicians. Screening was performed 'blind', i.e without prior knowledge of the origin of the samples to prevent bias in the interpretation of the results.

### *Assay and statistical analysis*

The solid phase, competitive RIA for human α-lactalbumin was performed as follows. Five micrograms of rabbit anti-mouse Ig (RAM Ig, DAKO Denmark) were incubated with 1 ml of a 10% (w/v) suspension of Cowan 1 strain *Staph. aureus* for 30 min at 4°C. The rabbit anti-mouse-*Staph. aureus* complex (RAMSA) was washed 3 times in 50 mM Tris/HCl, 0.6M NaCl, 0.5% Triton X 100, pH 8.3 and resuspended to a 10% (w/v) bacterial suspension. One ml (500 ng) of the monoclonal antibody specific for human α-lactalbumin (ET-1) was bound to one ml of RAMSA by incubation for 6 h at 4°C to produce RAMSET. Radioiodinated human α-lactalbumin tracer ($8 \times 10^5$ c.p.m. per 2 ml PBS, 0.2% BSA, specific activity 2,000 Ci mmol$^{-1}$) was added to the 2 ml of RAMSET and incubated overnight at 4°C with constant mixing.

Human sera was diluted 1/10 in PBS, 0.2% BSA containing 0.2% bovine α-lactalbumin and incubated overnight at 4°C to block natural antibody activity and to release bound human α-lactalbumin. The blocked sera and human α-lactalbumin standards (100 µl) were incubated with the tracer bound RAMSET (100 µl) in duplicate for 48 h at 4°C with constant mixing.

The coefficient of extinction of the human α-lactalbumin standards was $\epsilon_{0.1\%\ 280} = 1.8$. The bound counts were determined in the pellet after centrifugation at 10,000 *g* for 5 min. The amount of human α-lactalbumin in the serum samples were extrapolated from a standard curve plotted as B/Bo × 100 versus log human α-lactalbumin concentration where B is the bound counts and Bo is the maximum counts bound. Grouped data were evaluated statistically using the unpaired Student's *t* test.

### Results

Figure 1 shows the purity of the human α-lactalbumin analysed by two-dimensional gel electrophoresis and stained with Coomassie Blue; the protein is seen as a single acidic

**Figure 1** Two-dimensional gel electrophoresis showing the purity of human α-lactalbumin. The location of α-lactalbumin (mol. wt 14.5 kDa) on the gel is bracketed ([ ]). The spots at 92 kDa (phosphoylase B), 50 kDa (lgG heavy chain) and 25 kDa (lgG light chain) were included as markers.

spot of 14.5 kDa. Figure 2 is a typical standard curve for the human α-lactalbumin RIA. Intra- and inter-assay variations between several curves were compared and found to be insignificant, showing the reproducibility of the assay. The presence of bovine α-lactalbumin did not affect the standard curve significantly especially in the useful range from 10 to 30 ng ml.$^{-1}$

The mean level of α-lactalbumin found in 110 normal individuals (male and female) was 11 ± 2.3 ng ml$^{-1}$ (Table 1). Although there was variation between individuals, no statistically significant difference in circulating α-lactalbumin levels ($P<0.21$) was observed in age and sex matched controls. The cut off point distinguishing normal from elevated levels was arbitrarily set at 18 ng ml$^{-1}$ (mean + 3 s.d. = 17.9 ng ml$^{-1}$). Using this cut off and taking the levels for the third trimester of pregnancy as true positive and those of normal controls as

**Figure 2** Standard curve for human α-lactalbumin radioimmunoassay. ■——■, 100 ng bovine α-lactalbumin per tube; □——□, without bovine α-lactalbumin.

true negative, the sensitivity of the assay was 72% and the specificity 75%.

Levels of human α-lactalbumin were elevated in sera from patients with breast cancer (mean = 23.4 ± 5.6 ng ml$^{-1}$) (Table II). The highest levels were from patients with stage IV breast cancer. Eighty-five per cent of these patients have levels higher than the cut off and were matched only by those women in the last trimester of pregnancy. The elevated levels from patients with stage I–III breast cancer, were of the same order of magnitude as those from patients with gynaecological cancer. These levels were significantly higher ($P<0.001$) than those from controls, from women with benign breast tumours, and from patients with non-gynaecological cancers (Figure 3).

### Discussion

The measurement of human serum α-lactalbumin is often hampered by the presence of endogenous cross reacting antibodies to bovine α-lactalbumin (Woods & Heath, 1978; Zangerle & Franchimont, 1985). The present study is the first report of the application of a monoclonal antibody specific for human α-lactalbumin for a radioimmunoassay which has been developed to overcome this interference by the endogenous antibodies.

The levels of human α-lactalbumin in the sera of normal male and pregnant and non-pregnant females were initially assessed (Table I). All these sera showed detectable levels of α-lactalbumin. The reason for the occurrence of α-lactalbumin in normal male sera in not known. However, because the range of α-lactalbumin levels in the sera of males and non-pregnant females is narrow, they can be distinguished from the sera of individuals with elevated levels. The levels of human α-lactalbumin were approximately three times higher in pregnant females compared to males and non-pregnant females. The elevation of human α-lactalbumin levels in pregnancy is an expected finding and is consistent with previous reports (e.g. Martin *et al.*, 1980). These differences in the values obtained for pregnant females versus males and non-pregnant females validate the use of the assay as a quantitative test for circulating levels of human α-lactalbumin. For the purposes of the present study, the levels in males and non-pregnant females were taken as the baseline normal, negative controls and the levels in pregnant females as the positive controls.

Using the value of 18 ng ml$^{-1}$ (equivalent to the mean + 3 standard deviations of the negative controls) as the upper

**Figure 3** Circulating levels of human α-lactalbumin. Means (■) ± 2 standard deviations (bars) shown for each group. The arbitrary cut-off level of 18 ng ml$^{-1}$ was derived from the mean plus 3 standard deviations of the total male and female control groups. Gynaecological cancer comprised cancer of the ovary ($n = 70$), cervix ($n = 5$) and endometrium ($n = 5$). Miscellaneous cancers comprised cancer of the stomach ($n = 3$), leukaemia ($n = 8$), lung ($n = 5$), lymphomas ($n = 7$) and melanomas ($n = 2$).

**Table I** α-Lactalbumin levels in non-cancer sera

| Samples | Number Postive/Total | (%) | Serum α-lactalbumin mean (ng $ml^{-1}$) | (s.d.) |
|---|---|---|---|---|
| Third trimester pregnancy sera | 48/55 | (87.3) | 29.11 | (7.4) |
| Benign breast tumour sera | 3/22 | (13.6) | 13.33 | (4.8) |
| Normal male sera | 0/55 | (0) | 11.31 | (2.4) |
| Normal female sera | 0/55 | (0) | 10.75 | (2.2) |
| Normal sera (male and female) | | | 11.03 | (2.3) |

Cut off level of human α-lactalbumin = 18 ng $ml^{-1}$

**Table II** α-Lactalbumin levels in cancer sera

| Cancer | Number Postive/Total | (%) | Serum α-lactalbumin mean (ng $ml^{-1}$) | (s.d.) |
|---|---|---|---|---|
| Breast | | | | |
| Stage I | 8/20 | (40.0) | 16.18 | (3.8) |
| Stage II | 11/18 | (61.1) | 18.29 | (3.4) |
| Stage III | 15/27 | (55.5) | 17.73 | (3.9) |
| Stage IV | 28/32 | (87.5) | 29.35 | (7.2) |
| Gynaecological[a] | 56/80 | (70.0) | 19.14 | (6.7) |
| Miscellaneous[b] | 3/25 | (12.0) | 13.43 | (3.6) |

Cut off level of human α-lactalbumin = 18 ng $ml^{-1}$. [a]Gynaecological cancers include cancer of the ovary (51 positives), cervix (3 positives) and endometrium (2 positives). [b]Miscellaneous cancers include melanoma, lymphoma (2 positives), leukaemia, carcinoma of the stomach, colon and lung (1 positive).

limit of normal values, the results of the assays for the sera of patients with benign and malignant breast lesions showed that circulating α-lactalbumin levels were elevated in the sera of over 64% of patients with breast cancer. These results were significantly higher ($P<0.001$) than the values for α-lactalbumin found in the sera of women with benign breast tumours and in the controls (males and non-pregnant females). Significantly, the levels of α-lactalbumin in the sera of patients with benign breast lesions were not elevated. The elevated levels in breast cancer were due entirely to the high levels in patients with stage IV breast cancer with 85% of patients having raised levels (Tables I and II and Figure 3). The elevated levels in stage IV breast cancer were of the same order of magnitude as those observed for pregnant females. In the sera of patients with stage I, II and III breast cancer, the mean α-lactalbumin levels were also elevated, but only less than twice the mean of the normal level and the differences were not statistically significant ($P<0.2$). The levels of α-lactalbumin for patients with other non-breast cancers were not elevated (Table II and Figure 3). However, the levels in patients with gynaecological cancers were elevated.

It must be emphasised that the results obtained by this RIA may not represent the true serum α-lactalbumin concentrations. Although the human α-lactalbumin used in the standard curve was purified to homogeneity, there is the question of stability, heterogeneity of the protein in different populations and salt content which may affect the specific activity of the purified protein (Bangham & Cotes, 1971). Hence, the concentration of human α-lactalbumin reported here cannot be accurately compared with published results using other standards which will have different effective constituents.

Since the molecule is restricted strictly to breast tissue, the elevated serum levels found in patients with gynaecological cancers is an unexpected result. This appears to be a common occurrence among assays for other milk related antigens. RIAs of HMFG2 (Burchell *et al.*, 1984), DF3 antigen (Hayes *et al.*, 1985) and MAM6 antigen (Hilkens *et al.*, 1984) have also shown high levels of the respective antigens in ovarian carcinoma. It may be that gynaecological tumours to produce and secrete milk proteins (Ray *et al.*, 1986). However, the distinction between breast and gynaecological cancers is not a diagnostic problem.

The RIA for human α-lactalbumin at its present level of sensitivity is not suitable for widespread diagnostic screening, since the antigen is not elevated significantly in the sera of patients with early breast cancer. Before the value of the human α-lactalbumin assay as a breast cancer monitoring test can be established, more studies on sera from patients with breast and non-breast cancers need to be carried out. Furthermore, longitudinal and comparative studies with other markers should also be helpful in defining the predictive value of human α-lactalbumin levels for breast cancer, and in assessing whether these levels will be useful in monitoring breast cancer before and after treatment.

We thank Dr J. Tjandra and Dr M. Cauchi for the serum samples and Miss C. Jones for assistance in preparing the manuscript.

### References

BANGHAM, D.R. & COTES, P.M. (1971). Reference standards for radioimmunoassays. In *Radioimmunoassay Methods*, Kirkham, K.E. & Hunter, W.M. (eds) p. 345. Churchill Livingstone: Edinburgh.

BURCHELL, J., WANG, D. & PAPADIMITRIOU, J.T. (1984). Detection of the tumour associated antigens by the monoclonal antibodies HMFG-1 and HMFG-2 in serum of patients with breast cancer. *Int. J. Cancer*, **34**, 763.

FINDLAY, J.B.C. & BREW, K. (1972). The complete amino acid sequence of human α-lactalbumin. *Eur. J. Biochem.*, **27**, 65.

HAYES, D.F., SEKINE, H., OHNO, T., ABE, M., KEEFE, K. & KUFE, D.W. (1985). Use of a murine monoclonal antibody for detection of circulating plasma DF3 antigen levels in breast cancer patients. *J. Clin. Invest.*, **75**, 1671.

HILKENS, J., BUIJS, F., HILGERS, J. & 4 others (1984). Monoclonal antibodies against human milk fat globule membranes detecting differentiation antigens of the mammary gland and its tumours. *Int. J. Cancer*, **34**, 197.

KLEINBERG, D.L. (1975). Human α-lactalbumin: measurement in serum and in breast cancer organ cultures by radioimmunoassay. *Science*, **190**, 276.

MARTIN, R.H., GLASS, M.R., CHAPMAN, C., WILSON, G.D. & WOODS, K.L. (1980). Human α-lactalbumin and hormonal factors in pregnancy and lactation. *Clin. Endocrinol.*, **13**, 223.

O'FARRELL, P.H. (1975). High resolution two-dimensional electrophoresis of proteins. *J. Biol. Chem.*, **250**, 4007.

RAY, D.B., JANSEN, R.W., HORST, I.A., MILLS, N.C. & KOWAL, J. (1986). A complex non-coordinate regulation of alpha lactalbumin and 25K β-casein by corticosterone, prolactin and insulin in long term cultures of normal rat mammary cells. *Endocrinology*, **118**, 393.

SCHULTZ, G.S. & EBNER, K.E. (1977*a*). Measurement of α-lactalbumin in serum and mammary tumours of rats by radioimmunoassay. *Cancer Res.*, **37**, 4482.

SHULTZ, G.S. & EBNER, K.E. (1977*b*). α-Lactalbumin levels in human mammary tumours, sera and mammary cell lines. *Cancer Res.*, **37**, 4489.

THEAN, E.T. & TOH, B.H. (1989*a*). Production and characterization of murine monoclonal antibody to human α-lactalbumin. *Immunol. Cell Biol.*, **67**, 41.

THEAN, E.T. & TOH, B.H. (1989*b*). Western immunoblotting: Temperature-dependent reduction in background staining. *Anal. Biochem.*, **177**, 256.

WOODS, K.L. & HEATH, D.A. (1977). The radioimmunoassay of human lactalbumin. *Clin. Chim. Acta*, **78**, 129.

WOODS, K.L. & HEATH, D.A. (1978). The interference of endogenous antibodies to bovine lactalbumin in the radioimmunoassay of human lactalbumin in serum. *Clin. Chim. Acta*, **84**, 207.

ZAMIEROWSKI, M.M. & EBNER, K.E. (1980). A radioimmunoassay for mouse α-lactalbumin. *J. Immunol. Meth.*, **36**, 211.

ZANGERLE, P.F. & FRANCHIMONT, P. (1985). Validation of radioimmunoassay for human lactalbumin in the serum by testing the endogenous antibodies interference. *Eur. J. Cancer Clin. Oncol.*, **21**, 1201.


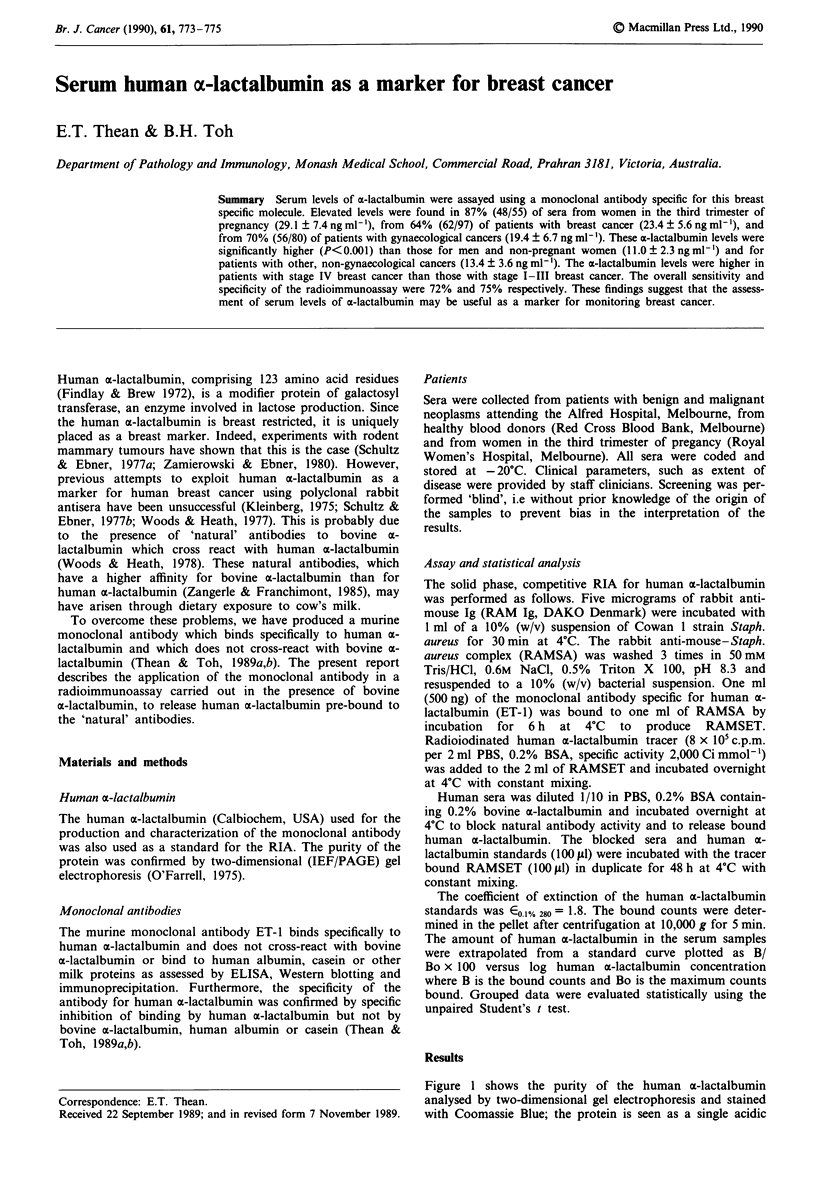

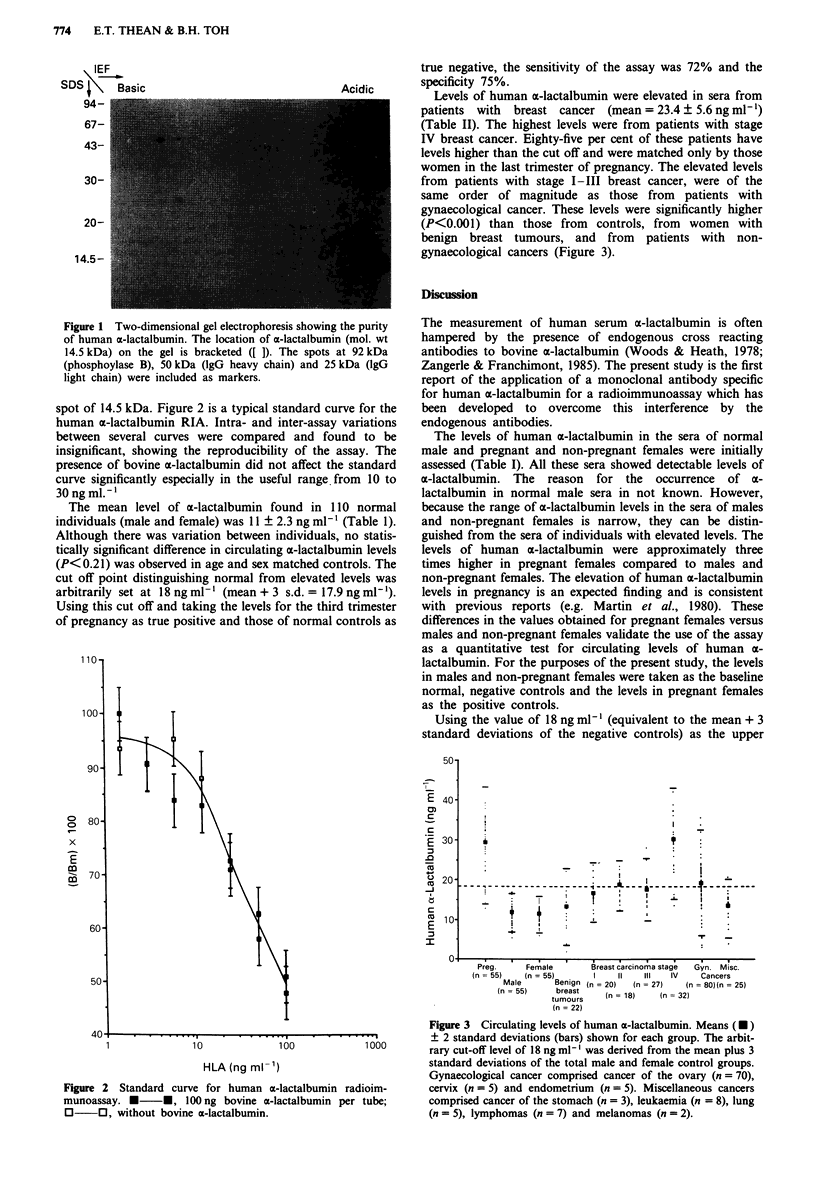

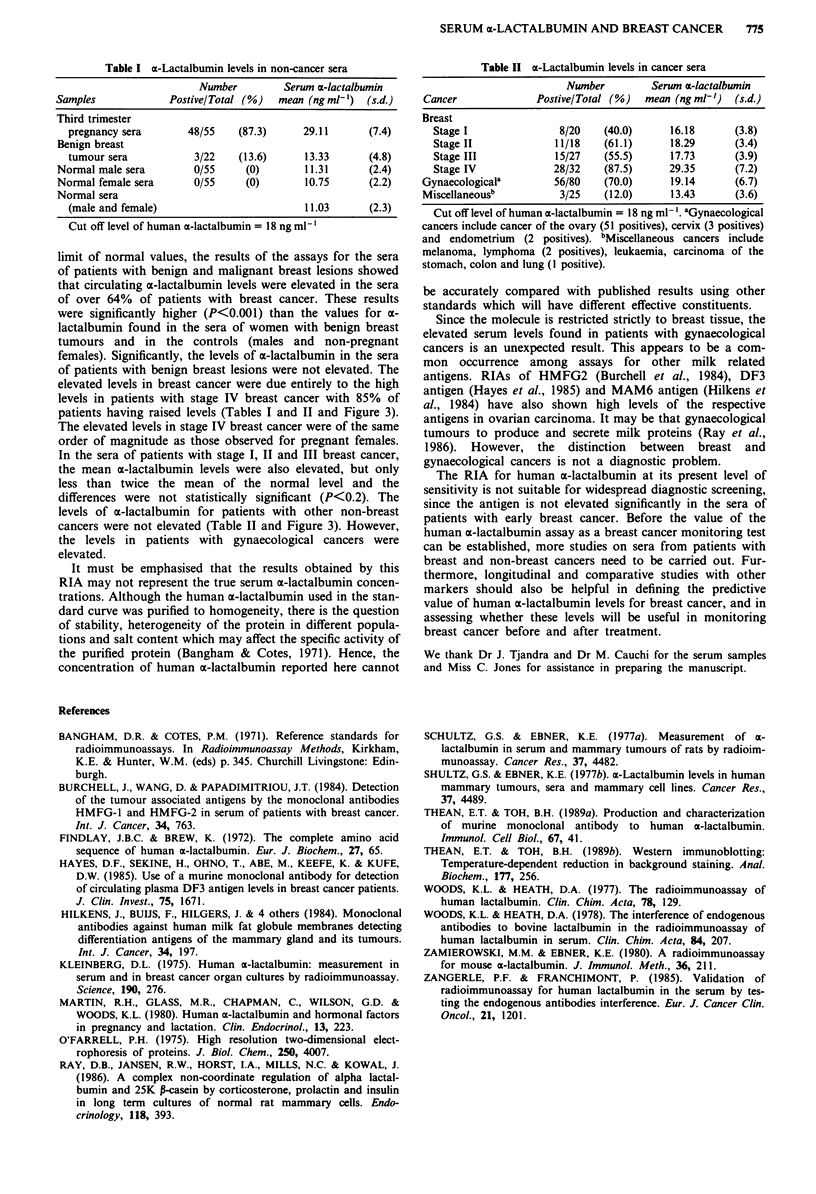


## References

[OCR_00416] Burchell J., Wang D., Taylor-Papadimitriou J. (1984). Detection of the tumour-associated antigens recognized by the monoclonal antibodies HMFG-1 and 2 in serum from patients with breast cancer.. Int J Cancer.

[OCR_00422] Findlay J. B., Brew K. (1972). The complete amino-acid sequence of human -lactalbumin.. Eur J Biochem.

[OCR_00426] Hayes D. F., Sekine H., Ohno T., Abe M., Keefe K., Kufe D. W. (1985). Use of a murine monoclonal antibody for detection of circulating plasma DF3 antigen levels in breast cancer patients.. J Clin Invest.

[OCR_00432] Hilkens J., Buijs F., Hilgers J., Hageman P., Calafat J., Sonnenberg A., van der Valk M. (1984). Monoclonal antibodies against human milk-fat globule membranes detecting differentiation antigens of the mammary gland and its tumors.. Int J Cancer.

[OCR_00438] Kleinberg D. L. (1975). Human alpha-lactalbumin: measurement in serum and in breast cancer organ cultures by radioimmunoassay.. Science.

[OCR_00443] Martin R. H., Glass M. R., Chapman C., Wilson G. D., Woods K. L. (1980). Human alpha-lactalbumin and hormonal factors in pregnancy and lactation.. Clin Endocrinol (Oxf).

[OCR_00448] O'Farrell P. H. (1975). High resolution two-dimensional electrophoresis of proteins.. J Biol Chem.

[OCR_00452] Ray D. B., Jansen R. W., Horst I. A., Mills N. C., Kowal J. (1986). A complex noncoordinate regulation of alpha-lactalbumin and 25 K beta-casein by corticosterone, prolactin, and insulin in long term cultures of normal rat mammary cells.. Endocrinology.

[OCR_00459] Schultz G. S., Ebner K. E. (1977). Measurement of alpha-lactalbumin in serum and mammary tumors of rats by radioimmunoassay.. Cancer Res.

[OCR_00464] Schultz G. S., Ebner K. E. (1977). alpha-Lactalbumin levels in human mammary tumors, sera, and mammary cell culture lines.. Cancer Res.

[OCR_00469] Thean E. T., Toh B. H. (1989). Production and characterization of murine monoclonal antibody to human alpha-lactalbumin.. Immunol Cell Biol.

[OCR_00474] Thean E. T., Toh B. H. (1989). Western immunoblotting: temperature-dependent reduction in background staining.. Anal Biochem.

[OCR_00483] Woods K. L., Heath D. A. (1978). The interference of endogenous antibodies to bovine lactalbumin in the radioimmunoassay of human lactalbumin in serum.. Clin Chim Acta.

[OCR_00479] Woods K. L., Heath D. A. (1977). The radioimmunoassay of human lactalbumin.. Clin Chim Acta.

[OCR_00488] Zamierowski M. M., Ebner K. E. (1980). A radioimmunoassay for mouse alpha-lactalbumin.. J Immunol Methods.

[OCR_00492] Zangerle P. F., Franchimont P. (1985). Validation of radioimmunoassay for human lactalbumin in the serum by testing the endogenous antibodies interference.. Eur J Cancer Clin Oncol.

